# Conserved Surface Accessible Nucleoside ABC Transporter Component SP0845 Is Essential for Pneumococcal Virulence and Confers Protection *In Vivo*

**DOI:** 10.1371/journal.pone.0118154

**Published:** 2015-02-17

**Authors:** Sneha Saxena, Naeem Khan, Ruchika Dehinwal, Ajay Kumar, Devinder Sehgal

**Affiliations:** Molecular Immunology Laboratory, National Institute of Immunology, Aruna Asaf Ali Marg, New Delhi, India; Centers for Disease Control & Prevention, UNITED STATES

## Abstract

*Streptococcus pneumoniae* is a leading cause of bacterial pneumonia, sepsis and meningitis. Surface accessible proteins of *S. pneumoniae* are being explored for the development of a protein-based vaccine in order to overcome the limitations of existing polysaccharide-based pneumococcal vaccines. To identify a potential vaccine candidate, we resolved surface-associated proteins of *S. pneumoniae* TIGR4 strain using two-dimensional gel electrophoresis followed by immunoblotting with antisera generated against whole heat-killed TIGR4. Ten immunoreactive spots were identified by mass spectrometric analysis that included a putative lipoprotein SP0845. Analysis of the inferred amino acid sequence of *sp0845* homologues from 36 pneumococcal strains indicated that SP0845 was highly conserved (>98% identity) and showed less than 11% identity with any human protein. Our bioinformatic and functional analyses demonstrated that SP0845 is the substrate-binding protein of an ATP-binding cassette (ABC) transporter that is involved in nucleoside uptake with cytidine, uridine, guanosine and inosine as the preferred substrates. Deletion of the gene encoding SP0845 renders pneumococci avirulent suggesting that it is essential for virulence. Immunoblot analysis suggested that SP0845 is expressed in *in vitro* grown pneumococci and during mice infection. Immunofluorescence microscopy and flow cytometry data indicated that SP0845 is surface exposed in encapsulated strains and accessible to antibodies. Subcutaneous immunization with recombinant SP0845 induced high titer antibodies in mice. Hyperimmune sera raised against SP0845 promoted killing of encapsulated pneumococcal strains in a blood bactericidal assay. Immunization with SP0845 protected mice from intraperitoneal challenge with heterologous pneumococcal serotypes. Based on its surface accessibility, role in virulence and ability to elicit protective immunity, we propose that SP0845 may be a potential candidate for a protein-based pneumococcal vaccine.

## Introduction

*Streptococcus pneumoniae* (also referred to as pneumococcus) is a major cause of life-threatening diseases such as pneumonia, bacteremia and meningitis. Pneumococcus is responsible for a significant amount of morbidity and mortality among children globally and particularly in developing countries. Pneumococcal disease caused an estimated 800000 deaths in children below 5 years of age [1]. Children less than 2 years of age, the elderly (> 65 years) and immunocompromised individuals are at high risk for pneumococcal infection. The rapid emergence of resistance to antimicrobials (e. g. penicillin, macrolides and cephalosporin) has complicated the global management of pneumococcal diseases [2].

The capsular polysaccharide that envelops pneumococci is its major virulence factor. Based on the capsular polysaccharide, pneumococci have been classified into over 90 serotypes. The prevalence of the disease-causing serotypes varies from region to region and by age. Recent data suggests that serotypes 1, 5, 6B, 14, 19F and 23F are the most prevalent serotypes globally [3]. Pneumococcal capsular polysaccharide (PCP) induces serotype-specific antibodies that activate and fix complement, and promote opsonization and phagocytosis of *S*. *pnuemoniae*.

The currently licensed pneumococcal vaccines are based on PCP. The 23-valent-polysaccharide (PPV23) vaccine formulation is a physical mixture of PCP from 23 different vaccine serotypes. Limited serotype coverage, poor immunogenicity in high-risk groups and serotype replacement limit the use of PPV23 vaccine. The 7-valent glycoconjugate (PCV7) vaccine is based on the chemical coupling of the capsular polysaccharide from various serotypes to CRM197, a genetically detoxified, cross-reactive mutant of diphtheria toxin. The PCV7 vaccine elicits higher antibody titers in infants, the elderly and in immunocompromised individuals than the PPV23 vaccine. In addition to inducing protection against invasive disease it also induces immunological memory and herd immunity [4]. Neither pneumococcal vaccine provides sufficient protection in populations where the dominant serotypes are other than those included in the currently licensed vaccines [5]. In recent years, replacement of vaccine serotypes by non-vaccine serotypes has also been reported [6–9].

New conjugate vaccines are being developed to increase serotype coverage. Recently, 10-valent and 13-valent glycoconjugate vaccine formulations have been licensed [10–13], and a 15-valent version is under development [14]. Glycoconjugate is prepared for individual serotype; increasing the number of serotypes enhances the cost, thus limiting its deployment in developing countries where vaccination is most needed. These concerns, combined with increasing antibiotic resistance, are driving search for an affordable 'universal' vaccine that confers serotype independent protection in all age groups.

In general, proteins that are antigenically conserved across epidemiologically relevant serotypes are expected to be more effective immunogens. In recent years, efforts have been made towards the development of protein-based pneumococcal vaccine. Conventional approaches have targeted single candidate proteins based on their roles in bacterial pathogenicity and physiology [5,14–17]. PspA [5,18] and histidine triad protein D have been taken to phase I trial [19,20]. In preclinical evaluation, Pht proteins (A, B, D and E) were shown to be protective against nasopharyngeal colonization and systemic infection in mouse models [21]. Comprehensive 'omics'-based approaches have been utilized for the identification of potential candidates for a protein-based pneumococcal vaccine [5,22–24]. Bioinformatics-based analysis of the whole genome was used to identify 6 proteins that protected mice against disseminated infection [25]. Ling et al used an immunoproteomic approach to screen cell wall enriched proteins and demonstrated that glyceraldehyde-3-phosphate dehydrogenase and fructose-bisphosphate aldolase conferred protection [26]. Giefing et al used display libraries and serum from convalescing patients to identified PcsB and StkP as vaccine candidates [27]. Moffit et al used proteomic analysis to identify conserved protein vaccine antigens that elicit T_H_17-dependent responses capable of preventing colonization [28].

The available data suggests that a single protein may not be sufficient to provide species-wide protection, and therefore combinations of different proteins are being tested [29–32]. The goal of this study was to identify a novel pneumococcal surface protein that can confer protective immunity. Here, we report the evaluation of the vaccine potential of SP0845, a novel protein antigen. We analyzed its physiological function, intra-species sequence conservation, surface accessibility, immunogenicity, role in virulence and its protective activity in a mouse model of sepsis. Our functional studies demonstrate that SP0845 is the conserved substrate-binding component of an ABC transporter that is involved in the uptake (or scavenging) of nucleosides. Further, our data show that SP0845 is essential for virulence and immunization with SP0845 protects mice from pneumococcal challenge. Our study suggests that SP0845 can serve as a candidate for a vaccine against *S*. *pneumoniae* infection.

## Materials and Methods

### Ethics statement

All animal experiments were conducted with the approval and following the guidelines of the Institutional Animal Ethics Committee of the National Institute of Immunology, New Delhi (IAEC#229/10). All efforts were made to minimize suffering.

### Pneumococcal strains and culture conditions

Pneumococci were maintained and heat-killed as described previously [33].

The pneumococcal strains ATCC 6301, ATCC 6303, ATCC 6305, ATCC 6314, ATCC 6319, ATCC 6323, ATCC 6326 and ATCC BAA-334 (referred to as TIGR4 in this study) were obtained from American Type Culture Collection (ATCC), USA. The corresponding serotypes according to ATCC are 1, 3, 5, 14, 19, 23, 26 and 4, respectively. D39 (NCTC 7466) and A66 (NCTC 7978) were sourced from National Collection of Type Cultures, United Kingdom, and are of serotypes 2 and 3, respectively. R36A (unencapsulated variant of D39) was procured from ATCC. The strains were chosen based on their serotype, virulence status in mouse models and the fact that the strains have been used by several investigators in the field to do similar *in vitro* or *in vivo* experiments.

### Mice and immunization

Mice were kept in a pathogen free facility with free access to food and water, with regulated daylight, humidity and temperature.

Six to eight week old female BALB/c mice were immunized with 10^8^ cfu of whole heat-killed TIGR4 or 25 μg of recombinant SP0845 (SP0845^1–350^ or SP0845^23–350^) formulated in 25 μg alum (Pierce, USA; Catalogue: 77161) subcutaneously followed by two booster shots on day 14 and 28.

Mice were bled from the retro-orbital venous plexus under ketamine/ xylazine anaesthesia to collect the preimmune (3 days prior to the first immunization) and hyperimmune anti-SP0845^23–350^ sera (at day 35).

### Two dimensional gel electrophoresis and mass spectroscopy

Pneumococcal surface associated proteins were extracted as described by Overweg et al [34]. Briefly, the cell envelope fraction was obtained by ultracentrifuging lysate from TIGR4 at 150000 × g for 1 h at 4°C. The pellet was washed with PBS to remove potential contaminating cytosolic proteins. The surface associated proteins were extracted with a buffer (150 mM NaCl / 10 mM MgCl_2_ / 10 mM Tris, pH 8) containing 1% 3-(N, N-dimethyl myristylammonio)propanesulfonate (SB14) with constant stirring at 4°C for 3–4 h. Proteins were precipitated with acetone and quantified using a Micro BCA protein assay kit (Thermo Scientific, USA; Catalogue number: PI-23235). Hundred micrograms were loaded on an Immobiline DryStrip gel (pI = 4–7, 13 cm) and resolved by isoelectric focusing using an Ettan IPGphor 3 system (GE Healthcare Biosciences, USA). The program used for separation was as follows: rehydration for 6 h, isoelectric focusing at 50 V for 5 h, 500 V for 2 h, 1000 V for 30 min, 2000 V for 30 min, 4000 V for 1 h and 5000 V for 4 h. The Immobilize Dry strip gel was equilibrated with dithiothreitol and iodoacetamide for 15 min each. The proteins were then separated on a 10% SDS-PAG (at 40 mA for 5–6 h) using SE 600 Ruby gel electrophoresis system (GE Healthcare Biosciences). The gel was either stained by Vorum’s silver staining method [35] or transferred to polyvinylidene fluoride (PVDF) membrane (MDI, India) and probed with polyclonal sera raised against whole heat-killed TIGR4 as described under the 'Western blot analysis' section for identifying immunoreactive proteins. Immunoreactive spots were excised from the silver stained gel and processed for peptide mass fingerprinting by matrix assisted laser dependent ionization time of flight (MALDI-TOF) or nano liquid chromatography mass spectrometry at the Institute of Molecular Medicine, New Delhi. The proteins were identified by searching an online database (www.matrixscience.com).

### Western blot analysis

BALB/c mice were injected with 10^6^ cfu of ATCC 6303 intraperitoneally. After 18 h, blood was collected from the retro-orbital venous plexus in a heparinized tube under ketamine/ xylazine anaesthesia. The mice were euthanized by cervical dislocation before removing the spleen. Red blood cells present in blood and splenocytes were lysed by treating with 0.15 M NH_4_Cl at room temperature for 10–15 min. NH_4_Cl-treated samples were centrifuged at 1200 × g for 5 min at 4°C to pellet the host cells. Pneumococci were pelleted from the supernatant by centrifuging at 5000 × g for 15 min at 4°C.

For immunoblot analysis, lysates were prepared by resuspending the pneumococcal cell pellet (obtained from *in vitro* grown culture, blood or spleen) in gel loading buffer and heating it at 100°C for 20 min. Samples resolved by two dimensional gel electrophoresis (pneumococcal surface associated proteins) or SDS-PAGE (purified recombinant protein and pneumococcal lysates) were transferred to PVDF or nitrocellulose (Bio-Rad Laboratories) membrane using a semi-dry transfer apparatus (Bio-Rad Laboratories). The membrane was blocked with 2% skimmed milk followed by 1 h incubations with primary antibody [polyclonal sera raised in mouse against whole heat-killed TIGR4 (diluted 1 in 1000) or anti-histidine affinity tag (diluted 1 in 1000) antibody (Sigma; Catalogue number: H1029) or mouse anti-SP0845 antibody (diluted 1 in 500)] and horse radish peroxidase conjugated goat anti-mouse Ig antisera (diluted 1 in 1000; BD Biosciences, USA; Catalogue number: 554002) as the secondary antibody at room temperature. The membrane was washed with PBS containing 0.05% Tween-20 (PBST) after each incubation step. The blot was developed with either enhanced chemiluminescence reagent (GE Healthcare Biosciences; Catalogue number: 32106) or 3, 3’-diaminobenzidine/ H_2_O_2_ as substrate. The chemiluminiscence was detected using an X-ray film (GE Healthcare Biosciences).

### Molecular cloning, expression and purification of recombinant SP0845

The subfragment corresponding to the codons 23 to 350 of the *sp0845* open reading frame was PCR amplified from TIGR4 genome using *Pfu* DNA polymerase (Strategene, USA; Catalogue number: 600140), and 5’-ggtaaccgctcttctcgtaa-3’ and 5’-ccccccaagcttttatttttcaggaacttttacgc-3’ (engineered HindIII site is underlined) as sense and antisense primers, respectively. The program used for amplification was as follows: denaturation at 95°C for 2 min followed by 25 cycles of denaturation for 1 min at 94°C, annealing for 1 min at 55°C and extension for 1 min at 72°C. There was a concluding extension step at 72°C for 7 min. The PCR amplified fragment was digested with HindIII (New England Biolabs, USA; Catalogue number: R0104S), cloned in the expression vector pQE-30Xa (Qiagen, Germany; Catalogue number: 33203) and transformed into *E*. *coli* strain XL-1 blue. The recombinant construct was confirmed by restriction digestion and DNA sequencing. For expressing the protein, the construct was transformed into *E*. *coli* strain SG13009 (Qiagen; Catalogue number: 34210). Protein expression was induced by addition of 1 mM isopropyl-β-D-thiogalactopyranoside and purified using Ni-NTA affinity column chromatography (Sigma; Catalogue number: P6611) followed by a DEAE sepharose anion-exchange chromatography (GE Healthcare Biosciences; Catalogue number: 17-0709-01). The purity of the recombinant SP0845 (referred to as SP0845^23–350^) preparation was assessed by SDS-PAGE.

The ORF encoding SP0845 (amino acids 1–350) was also amplified using 5’-atgaacaagaaacaatggctag-3’ and 5’-ccccccaagcttttatttttcaggaacttttacgc-3’ as sense and antisense primers, respectively. The amplicon was cloned, expressed and purified as described for SP0845^23–350^ and referred to as SP0845^1–350^.

### PCR amplification and sequencing of sp0845 homologues from various pneumococcal strains

Genomic DNA was isolated from various pneumococcal strains using a genomic DNA isolation kit (Qiagen; Catalogue number: 10243). The ORF encoding SP0845 (or its homologues) was amplified from pneumococcal genomic DNA using KAPA Hi-Fi DNA polymerase (Kapa Biosystems, USA; Catalogue number: KK2102). The PCR components and reaction conditions were the same as described above for SP0845. The resultant amplicon was purified using a gel extraction kit (Real Biotech Corporation, Taiwan; Catalogue number: YDF300) and sequenced. The DNA sequence data was analyzed using Sequencher version 4.6, (Gene Codes Inc., USA) and MacVector with assembler version 12.7.5 (MacVector Inc., USA), and the nucleotide sequences obtained were submitted to GenBank.

### Construction of spd_0739 deficient strain of D39 and its genetic complementation

A D39 mutant deficient in *spd_0739* (homologue of *sp0845*) was constructed by an in-frame replacement of *sp0845* with a kanamycin cassette using an overlap PCR strategy described previously [36,37]. The construct was introduced into pneumococci using competence-stimulating peptide as described previously [38]. The mutant was confirmed by colony PCR and nucleotide sequencing.

A shuttle expression vector pDC123 was used for genetic complementation of *spd_0739* deficient D39 [39]. The complete *spd_0739* open reading frame was PCR amplified from D39 genome using KAPA Hi-Fi DNA polymerase and primers DS_1051 (5'-ccccccagcgctaggaggaaacaaggaaatgaacaagaaacaatggctag-3') and DS_1052 (5'-ccccccagatctttatttttcaggaacttttacgc-3'). HaeII and BglII sites were incorporated in the primers DS_1051 and DS_1052, respectively to facilitate cloning. The expression vector pDC123 was digested with HaeII and BglII to remove the *phoZ* gene and clone HaeII-BglII digested *spd_0739* gene. The HaeII-BglII digested pDC123 was end-filled, polished and circularized (pDC123_DS). The pDC123_DS with or without insert was transformed in *E*. *coli* MC1061 for propagation. The recombinant plasmid was transformed in *spd_0739* deficient D39 using competence-stimulating peptide.

A *sp0845* deficient mutant of TIGR4 and a second independently generated *spd_0739* deficient mutant of D39 were generated as described above.

### 5-fluorouridine sensitivity and competition assay

To check for sensitivity to the toxic nucleoside analog 5-fluorouridine (5FU) wildtype D39, *spd_0739 deficient* and genetically complemented D39 mutant strains were grown in THY in the absence or presence of 0.1 mM 5FU. The absorbance of the pneumococcal cultures was recorded at 620 nm after 6 h using a spectrophotometer (GE healthcare Life Sciences). The 5FU sensitivity assay was also performed with TIGR4 and its *sp0845* deficient mutant, and an independently constructed D39 strain deficient in *spd_0739* and its wildtype counterpart D39.

For competition assay, wildtype D39 was grown for 6 h in presence of 0.05 mM 5FU and ten times excess (0.5 mM) of ribonucleosides (cytidine, uridine, guanosine, adenosine and inosine), deoxyribonucleosides (deoxycytidine, deoxyuridine, deoxyguanosine, deoxyadenosine and thymidine), pyrimidine nucleobases (cytosine, uracil and thymine), purine nucleobase (adenine, xanthine and hypoxanthine) or sugar (ribose). The optical density of the cultures was recorded at 620 nm after 6 h. The experiment was performed in duplicates.

### Immunofluorescence microscopy

Immunofluorescence microscopy was performed as described by Johnston et al with minor modification [40]. Briefly, pneumococci were harvested from mid-logarithmic phase (OD_600_ = 0.4–0.6) culture by centrifuging at 5000 × g for 15 min followed by washing with PBS. The pellet were resuspended in PBS containing 2% BSA and incubated for 30 min at 37°C. After three washes with PBS, pneumococci were incubated with primary antibody (mouse anti-SP0845^23–350^ or normal mouse sera diluted 1 in 100) for 1 h at 37°C. Following three washes with PBS, pneumococci were incubated with secondary antibody [F(ab')_2_ fragment phycoerythrin conjugated goat anti-mouse IgG + IgM (H + L) antibody (diluted 1 in 500; Jackson ImmunoResearch Laboratories, USA; Catalogue number: 115–116–068)] for 1 h at 4°C in the dark. After three washes with PBS, pneumococci were resuspended in 3% paraformaldehyde for 10 min at 4°C and applied as a smear on a polylysine coated slide (Thermo Shandon, USA). The slide was air-dried and one drop of antifade reagent (Bio-Rad Laboratories) was added before applying the coverslip. The slide was observed and photographed using a fluorescence microscope at a magnification of 1000X.

### Flow cytometry

The surface expression of SP0845 (or its corresponding homologue) was analyzed for *S*. *pneumoniae* strains TIGR4, ATCC 6303, ATCC 6314, D39, R36A (unencapsulated version of D39), and D39 and TIGR4 derived strains using flow cytometry. Mid-logarithmic phase (OD_600_ = 0.4, ~10^7^ cfu) pneumococcal cells were incubated with either mouse preimmune or mouse anti-SP0845^23–350^ sera (diluted 1 in 200 in PBS-0.5% BSA) for 1 h at room temperature. Pneumococci were washed with PBS and incubated with FITC-conjugate F(ab')_2_ fragment goat anti-mouse IgG + IgM (H + L) antibody (diluted 1 in 200; Jackson ImmunoResearch Laboratories; Catalogue number: 115–096–068) at room temperature for 1 h. After washing, the cells were fixed with 2% paraformaldehyde for 10 min at 4°C and run on a flow cytometer (FACS caliber, Becton-Dickinson, USA). The results were analyzed by FlowJo software version 10.0.6 (http://www.flowjo.com, USA).

### ELISA

Ninety six well microtiter plate (Greiner Bio-one, Germany) was coated overnight at 4°C with SP0845^23–350^ (50 μl of 5 μg/ml per well) in 100 mM carbonate-bicarbonate buffer, pH 9.5. The plate was washed with PBST and blocked with PBS containing 2% BSA for 1 h at 37°C. After washing the plate with PBST, serum samples (normal mouse or anti-SP0845^23–350^ polyclonal sera pooled from 12 mice hyperimmunized with recombinant SP0845^23–350^) serially diluted in PBS containing 0.5% BSA) were added in duplicate and incubated for 1 h at 37°C. The plate was washed with PBST and incubated with alkaline phosphatase conjugated goat anti-mouse sera specific for total IgG (SouthernBiotech, USA; Catalogue number: 1030–04), IgG1 (Catalogue number: 1070–04), IgG2a (Catalogue number: 1080–04), IgG2b (Catalogue number: 1090–04) or IgG3 (Catalogue number: 1100–04) (diluted 1 in 2000) as secondary antibody followed by incubation for 1 h at 37°C. The plate was washed with PBST and colour was developed using p-nitrophenol phosphate as substrate. The absorbance was recorded at 405 nm and plotted as a function of dilution factor using SigmaPlot 11 software. SP0845-specific serum antibody endpoint titer was determined using two times the optical density obtained for the preimmune sera (diluted 1 in 100) as the cut-off value.

### Blood bactericidal assay

The murine blood bactericidal assay was adapted from Briles et al [41]. Briefly, peripheral blood was collected from CBA/N mice under terminal ketamine/ xylazine anaesthesia by cardiac puncture. Recombinant hirudin from yeast (70 U per ml blood) was used as an anticoagulant. Pneumococcal cells (100–150 cfu in 10 μl) from strains TIGR4, ATCC 6303, ATCC 6314 and D39 were incubated with 220 μl of hirudinized murine peripheral blood in the presence of 20 μl of either mouse pre-immune or mouse anti-SP0845^23–350^ sera. The samples were incubated at 37°C for 3 h with rotation. The surviving pneumococci were enumerated by plating serial dilutions (in duplicate) on TSA plates.

### *In vivo* virulence studies

Six to eight week old BALB/c mice (8 mice per group) were injected intraperitoneally with either D39 (10^5^ cfu; ~100 times LD_50_), *spd_0739* deficient D39 (10^5^ cfu), TIGR4 (10^6^ cfu; ~5000 times LD_50_) or *sp0845* deficient TIGR4 (10^6^ cfu) suspended in sterile PBS. The mice were monitored for survival every 12 h. The mice that were alive at the end of the 7 day observation period were euthanized by cervical dislocation.

### Active mouse protection assay

Groups of twelve 6–8 wk old female BALB/c mice were subcutaneously administered either 25 μg of SP0845^1–350^, SP0845^23–350^ or PspA^3–286^ from R36A [33] formulated in 25 μg of alum or PBS formulated in 25 μg of alum (given to control mice). One week after the second booster (day 35), mice were challenged intraperitoneally with ATCC 6303 (10^5^ cfu; about one log greater than 50% lethal dose) or ATCC 6314 (5 x 10^6^ cfu). The pneumococcal challenge dose was arrived at based on pilot experiments and literature. Survival of the mice was monitored every 12 h for 21 days post-challenge. At the end of day 21, all surviving animals were euthanized by cervical dislocation.

The survival experiment was performed in accordance with the practices reported in recent studies on vaccine efficacy trials, infectious disease studies, microorganism virulence studies and animal studies of chronic infectious diseases [42–51]. In these studies, mortality was used as an endpoint (with no indication of use of humane euthanasia). We used death as the endpoint as early, reliable and unbiased biomarkers that can be used as indicators of disease severity are lacking. There is difficulty in reliably differentiating mice that will die from those which will recover, despite showing clinical signs precluding use of humane euthanasia. In addition, interpretation of clinical signs can itself be subjective in the absence of quantifiable indicators of disease severity. In our survival experiments, pain-relieving procedures could not be used because such measures may compromise the experimental integrity and validity of the study.

Investigators are strongly encouraged to carry out an exhaustive review of scientific literature pertaining to the pathogen of interest and experimental setup before undertaking any investigations that use death as an endpoint.

### Statistical analysis

Statistical analysis was carried out using SigmaPlot 11 and GraphPad Prism 6 software. The 5FU sensitivity and blood bactericidal assay data was analyzed by Student's unpaired t test. One-way ANOVA analysis was performed to determine the statistical significance of the data obtained from the competition assay and flow cytometry based assessment of the surface expression of SPD_0739 on D39 and its mutants. The *in vivo* virulence and active mouse protection data was assessed for statistical significance by carrying out Kaplan-Meier survival analysis (log rank test). *p* values ≤ 0.05 were considered statistically significant. *, *p* ≤ 0.05; **, *p* < 0.01; ***, *p* < 0.001; ****, *p* < 0.0001

### Nucleotide sequence accession numbers

The nucleotide sequences described in this study have been assigned GenBank accession numbers: HM775954 (ATCC 6301), HM775955 (ATCC 6303), HM775956 (ATCC 6305), HM775957 (ATCC 6314), HM775958 (ATCC 6319), HM775959 (ATCC 6323) and HM775960 (ATCC 6326).

## Results

### Identification of immunoreactive surface associated proteins from *S*. *pneumoniae*

**Fig 1:**
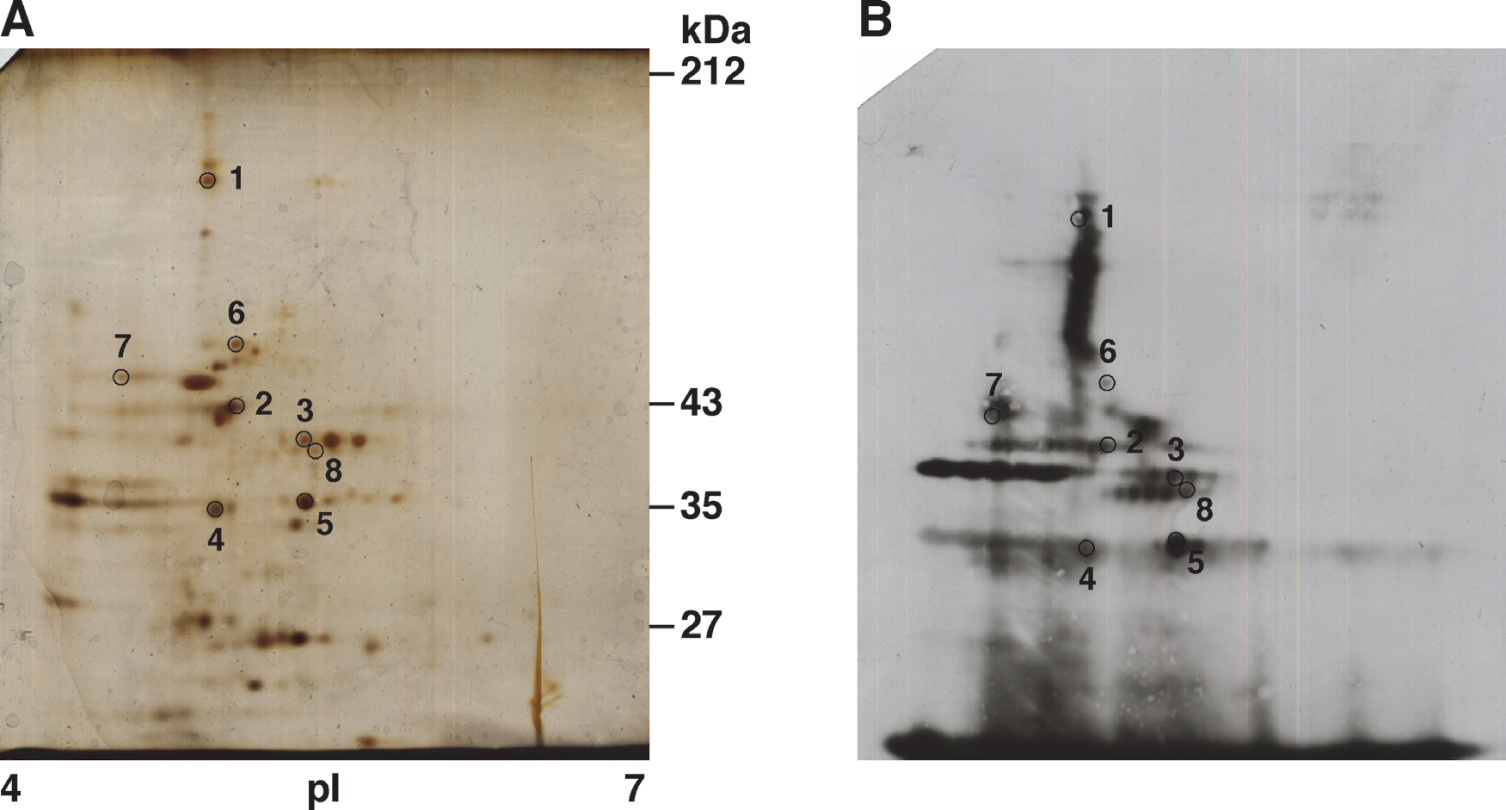
Identification of immunoreactive proteins from pneumococcal cell envelope fraction. The SB14 solubilized surface associated proteins of TIGR4 were resolved by 2D gel electrophoresis. The gel was either silver stained (A) or transferred to PVDF membrane, immunoblotted with polyclonal sera raised against whole heat-killed TIGR4 and developed using enhanced chemiluminescence reagent as substrate (B). The immunoreactive spots analyzed from the gel shown in panel A are numbered 1 through 8 (circled) and listed in Table 1. The molecular mass marker (in kDa) is shown to the right of panel A. The proteins were resolved by isoelectric focusing in the pH range 4 to 7 as marked in panel A.

In order to identify a surface associated protein that could have vaccine potential, soluble cell envelope fraction was resolved by 2D gel electrophoresis (Fig. 1A) and immunoblotted with mouse hyperimmune sera generated against whole heat-killed TIGR4 (Fig. 1B). The blot revealed many immunoreactive spots. Based on the physical separation of the protein spots on the silver-stained gel (Fig. 1A) and reactivity with polyclonal sera raised against whole heat-killed TIGR4 (Fig. 1B), we selected 8 spots (Fig. 1). Two additional spots (spot number 9 and 10 in Table 1) were selected from an independent experiment done under similar experimental conditions (data not shown). The spots were excised from the silver stained gel and analyzed by MALDI-TOF or nano liquid chromatography mass spectrometry. The proteins identified included some previously characterized pneumococcal proteins such as PspA (SP0117) [18], phosphoglycerate kinase (SP0499) [26], glyceraldehyde-3-phosphate dehydrogenase (SP2012) [26], maltose/ maltodextrin binding protein (SP2108) [52] and chaperone protein dnaK (SP0517) [26]. The uncharacterized proteins included a putative lipoprotein SP0845, PTS mannose specific IIAB component (SP0284), translation elongation factor tu (SP1489), peptide methionine sulphoxide reductase (SP1359) and trigger factor (SP0400). In this report, we evaluate the vaccine potential of SP0845. SP0845 was selected as it was predicted to be a lipoprotein. In pathogenic bacteria, lipoproteins have been demonstrated to play an important role in adhesion to host cells and colonization, and function as components of transport systems (involved in uptake of nutrients like sugars and metal ions) and virulence factors [53,54]. Lipoproteins have been demonstrated to have vaccine potential [15]. In addition, SP0845 has previously not been studied for its protective activity.

**Table 1:** Immunoreactive pneumococcal proteins identified in this study by mass spectrometry.

Spot no.^*a*^	ORF annotation^*b*^	Protein annotation	No. of matching peptides	Sequence coverage (%)^*c*^	Top MASCOT score^*d*^	MASCOT score cut off^*e*^
1	SP0117	Pneumococcal surface protein A	9	8	91	44
2	SP0499	Phosphoglycerate kinase	12	42	104	67
3	SP2012	Glyceraldehyde-3-phosphate dehydrogenase	11	20	79	44
4	SP0845	Putative lipoprotein	13	52	127	67
5	SP0284	PTS mannose specific IIAB component	24	32	537	29
6	SP1489	Translation elongation factor tu	6	14	199	45
7	SP2108	Maltose/maltodextrin binding protein	6	15	266	44
8	SP1359	Peptide methionine sulphoxide reductase	5	12	138	45
9	SP0400	Trigger factor	3	7	89	29
10	SP0517	Chaperone protein dnaK	5	5	60	29

^*a*^ For spot numbers 1 through 8 refer to Fig. 1. Spot number 9 and 10 were identified from an independent experiment performed under similar experimental conditions.

^*b*^ Gene and protein annotations are according to the genome sequence of *S*. *pneumoniae* TIGR4 (GenBank accession no. AE005672). ORF, open reading frame.

^*c*^ Percentage of total protein sequence covered by the experimentally detected peptides.

^*d*^ Identification probability of the fragment match.

^*e*^ MASCOT scores greater than the cut-off value were considered statistically significant (*p* ≤ 0.05).

### Molecular cloning, heterologous expression and purification of recombinant SP0845

**Fig 2:**
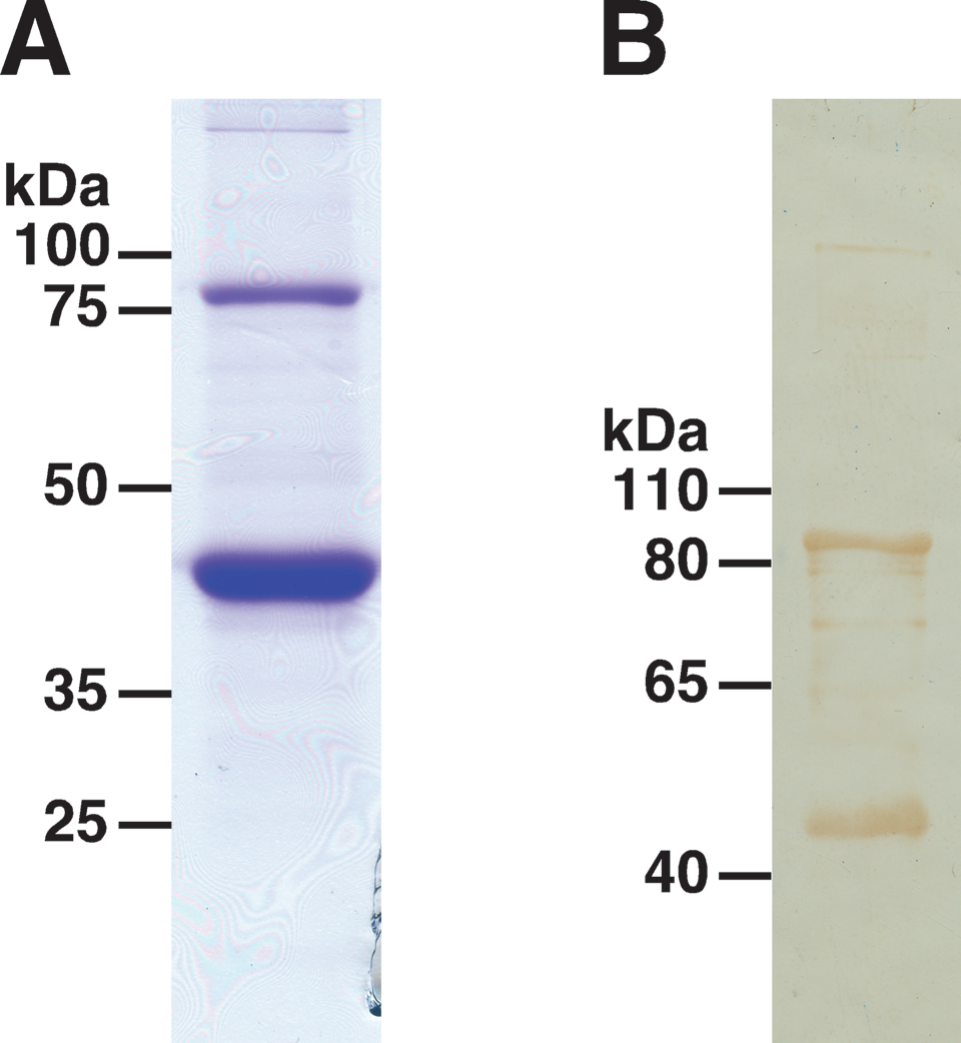
Purification and immunoblotting of SP0845. SP0845^23–350^ was expressed in *E*. *coli* and purified using Ni-NTA affinity and DEAE sepharose anion-exchange chromatography. The purified recombinant protein was resolved on 12% SDS-PAG and stained with Coomassie brilliant blue R250 (A) or transferred to nitrocellulose membrane and visualized using anti-histidine affinity tag antibody followed by horseradish peroxidase conjugated goat anti-mouse Ig as secondary antibody and 3, 3' diaminobenzidine/ H_2_O_2_ as substrate (B). The molecular mass marker (in kDa) is shown on the left of the panels.

The subfragment corresponding to amino acids 23–350 of SP0845 (SP0845^23–350^) was PCR amplified from TIGR4 genomic DNA. The amplicon was cloned, expressed and purified as an N-terminal histidine tagged recombinant fusion protein. Purified SP0845^23–350^ was analyzed by SDS-PAGE (Fig. 2A) and immunoblotted with anti-histidine affinity tag antibody (Fig. 2B). The predicted size of SP0845^23–350^ is 39.5 kDa that includes a 2.5 kDa contribution from the vector encoded N-terminal histidine tag. SDS-PAGE and immunoblot analysis identified two bands, one at the expected size of ~40 kDa and the other at ~80 kDa. Peptide mass fingerprinting and N-terminal sequencing confirmed that both entities were indeed SP0845^23–350^ indicating that the ~40 kDa and ~80 kDa molecular species represent monomer and dimer of SP0845^23–350^, respectively. In addition to SP0845^23–350^, a version corresponding to 1–350 amino acids of SP0845 (SP0845^1–350^) was expressed and purified. Like SP0845^23–350^, SP0845^1–350^ also showed the monomer-dimer pattern on SDS-PAG (data not shown). A similar monomer-dimer pattern has been reported in the case of the recombinant substrate-binding protein of another ABC transporter [55].

### SP0845 is highly conserved across pneumococcal strains

To assess the level to which SP0845 is conserved across strains the ORF encoding SP0845 was PCR amplified using genomic DNA from pneumococcal strains ATCC 6301, ATCC 6303, ATCC 6305, ATCC 6314, ATCC 6319, ATCC 6323 and ATCC 6326, and the resultant products were sequenced. Alignment of the deduced amino acid sequence of the mature SP0845 (amino acid residues 22–350) from TIGR4 with the homologues present in these strains, as well as with the 28 pneumococcal strains for which complete genome sequence information was available in NCBI at the time of analysis, indicated that SP0845 is highly conserved with an amino acid identity of >98%. We observed some variability at 9 (residues 30, 55, 64, 110, 114, 115, 116, 295 and 300 of the mature protein) out of 329 (2.73%) amino acid positions. The alignment further revealed that SP0845 exists as 8 distinct alleles (S1 Table). Allele 4 (20/36, 55.55%) and 7 (8/36, 22.22%) were the most commonly observed alleles in our data set. The fact that SP0845 was present in all the 36 strains analyzed suggested that it could be playing an important role in pneumococcal biology. The amino acid identity of SP0845 with homologues present in related species *Streptococcus mitis*, *Streptococcus oralis*, *Streptococcus mutans*, *Streptococcus agalactiae*, *Streptococcus suis and Streptococcus pyogenes* is 98, 95, 70, 68, 67 and 65%, respectively.

We looked for possible orthologue(s) of SP0845 in humans by searching the *Homo sapiens* non-redundant database at NCBI using DELTA-BLAST (default settings) with the deduced amino acid sequence of mature SP0845 as the query sequence. The top hit (metabotropic glutamate receptor 4) showed an amino acid identity of 10.6% with a query sequence coverage of 51% and an expect value of 4e-04. Homology values in this range are generally not considered significant. Thus, it is highly likely that SP0845 would induce high antibody titers that would not cross-react with human antigens when used as a vaccine.

### Bioinformatic analysis of SP0845

We used *in silico* tools to predict the function of SP0845. The SP0845 protein has a ‘lipobox’ containing 22 amino acid signal peptide at its N-terminus, a feature characteristic of bacterial lipoproteins [56,57]. The Cys residue at position 22 serves as the site for the transfer of diacylglyceryl moiety. A search of the Conserved Domain Database revealed the presence of PBP1_BmpA_PnrA-like domain (cd06354) in the 39–320 amino acid region. A search of the Protein Data Bank revealed structural similarity to PnrA, a purine nucleoside receptor from the bacterial pathogen *Treponema pallidum* [58]. PnrA appears to be an ABC type nucleoside transporter. SP0845 shares 36% amino acid sequence identity with PnrA. Analysis of the 3 dimensional structure of PnrA complexed with inosine (PDB ID: 2FQW), guanosine (2FQX) and adenosine (2FQY) showed that 10 out of the 12 (Asp 47, Ser 48, Phe 56, Asn 57, Asp 128, Met 178, Phe 206, Val 230, Gly 232, Val 257, Asp 258 and Lys 280) putative ligand-contacting residues are identical in SP0845 (S1 Fig.). PnrA ligand-contacting residues Ser 48 and Met 178 were substituted in SP0845 by Thr and Ile, respectively. Analysis of the TIGR4 genomic region surrounding *sp0845* indicated that adjacent genes are either components of ABC transporter [ATPase (*sp0846*) and permeases (*sp0847* and *sp0848*)] or are involved in nucleoside metabolism [pyrimidine nucleoside phosphorylase (*sp0842*), deoxyribose-phosphate aldolase (*sp0843*) and cytidine deaminase (*sp0844*)]. Our reverse transcription-polymerase chain reaction based operon analysis demonstrated that *spd_0739* (the D39 homologue of *sp0845*) is cotranscribed with *spd_0735*, *spd_0736*, *spd_0737*, *spd_0738*, *spd_0740*, *spd_0741* and *spd_0742* (data not shown). A comparison with *S*. *mutans* UA159 indicated that *smu_1124*, *smu_1123* and *smu_1122* are TIGR4 homologues of *sp0842*, *sp0843* and *sp0844*, respectively. Similarily, *smu_1121c*, *smu_1120*, *smu_1119c* and *smu_1118c* are homologues of *sp0845*, *sp0846*, *sp0847* and *sp0848*, respectively. In *S*. *mutans*, the proteins encoded by these genes are involved in the uptake and metabolism of nucleosides [59]. The bioinformatic analysis suggests that SP0845 is likely to be the substrate-binding component of an ABC transporter involved in the uptake of nucleosides in *S*. *pneumoniae*.

### SP0845 is involved in import of nucleosides and its absence renders pneumococci avirulent

To study the role of SP0845 (and its homologues) in pneumococcal physiology, a *spd_0739* (homologue of *sp0845*) deficient mutant of D39 was constructed. It was genetically complemented with a *spd_0739* expressing plasmid construct. To check for the expression of SPD_0739, lysates from wild type, *spd_0739* deficient mutant, genetically complemented or vector transformed mutant strains of D39 were resolved by SDS-PAGE. The observed profiles were indistinguishable (data not shown). The immunoblot analysis using anti-SP0845 sera indicated that SPD_0739 was expressed in wildtype D39 and genetically complemented D39 mutant as indicated by the presence of a band at the expected size (~40 kDa; S2A Fig.). The expression level of SPD_0739 in the wildtype and genetically complemented D39 mutant was comparable. As expected no band was observed in lysates from *spd_0739* deficient and vector transformed D39 mutant strains suggesting they are devoid of SPD_0739 protein. The expression of another lipoprotein PpmA in the wildtype, *spd_0739* deficient, genetically complemented and vector transformed D39 strains was comparable (S2B Fig.). The surface expression of SPD_0739 was confirmed by flow cytometry (S2C Fig.). A TIGR4 strain deficient in SP0845 and a second independently constructed mutant of D39 deficient in *spd_0739* was generated and confirmed as described above.

**Fig 3:**
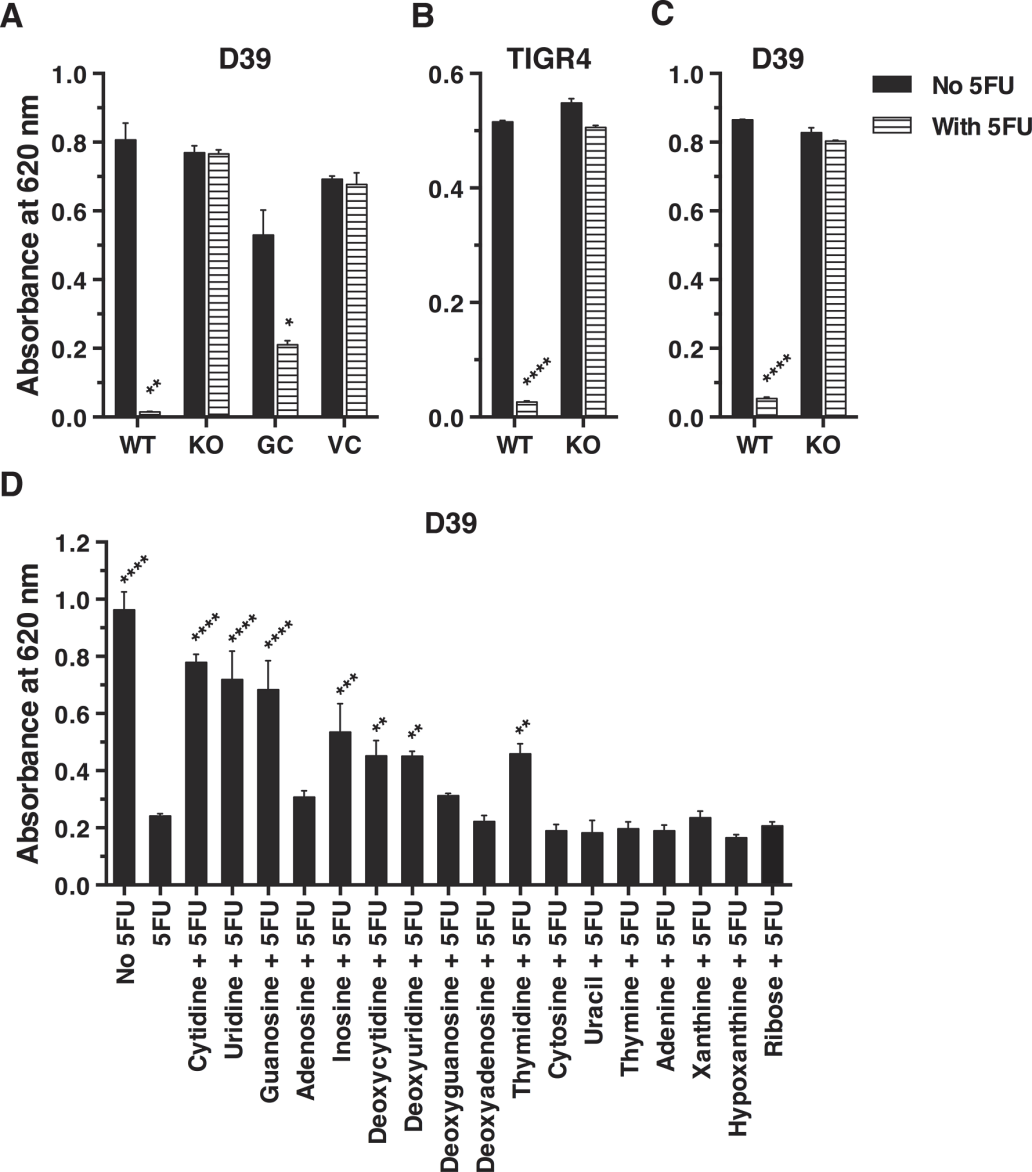
SPD_0739 (homologue of SP0845) is involved in nucleoside import by pneumococci. (A) Wildtype (WT) D39, *spd_0739* deficient (KO), *spd_0739* deficient strain genetically complemented with *spd_0739* (GC) and *spd_0739* deficient strain transformed with pDC123_DS (vector control; VC) were propagated either in the absence or presence of 0.1 mM 5-fluorouridine (5FU). (B) Wildtype TIGR4 and its *sp0845* deficient derivative were propagated either in the absence or presence of 0.1 mM 5FU. (C) Wildtype D39 and a second independently generated *spd_0739* deficient mutant were grown either in the absence or presence of 0.1 mM 5FU. The absorbance of the pneumococcal cultures was recorded at 620 nm after 6 h. For panels A, B and C the statistical significance was determined using the Student's t test with the corresponding 'No 5FU' sample as the reference. (D) Wildtype D39 was grown in presence of 0.05 mM 5FU and the indicated solutes at a concentration of 0.5 mM. Six hours later the absorbance was recorded. Wildtype D39 in the absence of 5FU served as the positive control. Error bars represent mean ± SD. The data presented is a representative of three independent experiments each performed in duplicates. One-way ANOVA analysis was performed using D39 sample with 5FU in the absence of any competing solute as the reference.

In order to decipher the functional role of SP0845, 5FU, a toxic nucleoside analog was added in the culture medium. The growth of wild type, *spd_0739* deficient, genetically complemented and vector transformed D39 strains was monitored spectrophotometrically at 6 h post-inoculation (Fig. 3A). The results demonstrated that wild type D39 was highly sensitive to 5FU as growth was completely abolished in its presence. On the other hand, *spd_0739* deficient D39 mutant was resistant to 5FU as growth was not retarded in its presence. Genetic complementation of the *spd_0739* deficient mutant with *spd_0739* restored the sensitivity towards 5FU to a significant extent but not to the wildtype level, confirming that the sensitivity to 5FU was specifically due to SPD_0739. *spd_0739* deficient D39 mutant transformed with vector was resistant to 5FU. The data indicates that 5FU is mainly taken up by pneumococci via SPD_0739. This was confirmed for *sp0845* deficient TIGR4 (Fig. 3B) and a second independently generated *spd_0739* deficient mutant of D39 (Fig. 3C).

To identify the natural ligands of SPD_0739, a competition assay was performed wherein various ribonucleosides (cytidine, uridine, guanosine, adenosine and inosine), deoxyribonucleosides (deoxycytidine, deoxyuridine, deoxyguanosine, deoxyadenosine and thymidine), pyrimidine nucleobases (cytosine, uracil and thymine), purine nucleobase (adenine, xanthine and hypoxanthine) and sugar (ribose) were allowed to compete with 5FU. The sensitivity of wildtype D39 strain to 5FU is expected to reduce if the added solute competes for transport via SPD_0739. The results indicated that cytidine, uridine, guanosine and inosine were competing better with 5FU than other solutes tested as the growth of wildtype D39 was better in their presence. Deoxycytidine, deoxyuridine and thymidine also recovered the growth of pneumococci but to a lesser extent suggesting that they compete less efficiently with 5FU. None of the nucleobases or sugar tested conferred resistance to 5FU toxicity. These data indicate that SPD_0739 interacts with nucleosides and is the substrate binding component of a multi-specificity nucleoside ABC transporter. This is the first experimental demonstration of nucleoside transport by an ABC transporter in *S*. *pneumoniae*.

In order to assess the possible role of SP0845 in pneumococcal virulence, groups of 8 mice were challenged intraperitoneally with 10^6^ cfu of wildtype TIGR4 or its *sp0845* deficient mutant. In a second experiment, mice were challenged with 10^5^ cfu of wildtype D39, *spd_0739* deficient mutant of D39 or a second independently constructed strain of D39 that lacks *spd_0739*. In both experiments, all the mice in the group challenged with either wildtype TIGR4 or D39 died within 48 h following challenge. All the mice challenged with *sp0845* deficient TIGR4, *spd_0739* deficient mutant of D39 or a second independently constructed D39 strain deficient in *spd_0739* survived for the duration (7 days) of the experiment (*p* < 0.0001; log rank test test). These data suggest that absence of SP0845 (or its homologue) renders *S*. *pneumoniae* avirulent.

### SP0845 is expressed in *in vitro* grown pneumococci and during infection

**Fig 4:**
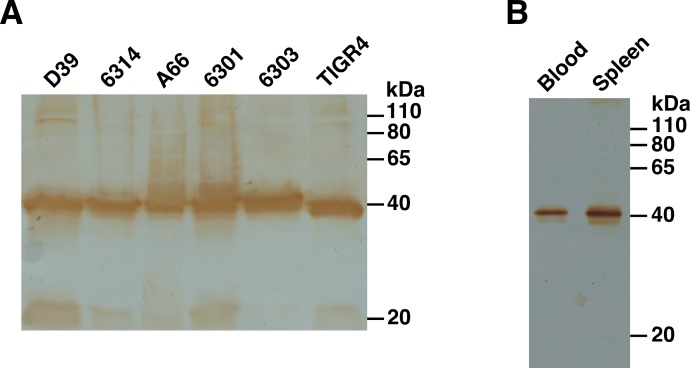
SP0845 protein is expressed by pneumococcal strains under *in vitro* and *in vivo* conditions. (A) Lysates were prepared (starting with mid-logarithmic phase cultures with similar optical density) from the indicated pneumococcal strains and resolved on a 12% SDS-PAG followed by transfer to nitrocellulose membrane. The blot was probed with mouse anti-SP0845^23–350^ polyclonal sera followed by horseradish peroxidase conjugated goat anti-mouse Ig antisera as the secondary antibody. 3, 3' Diaminobenzidiene along with H_2_O_2_ was used as the substrate. (B) Immunoblot analysis done using lysates prepared from pneumococci isolated from blood and spleen 18 h after infecting BALB/c mice with 10^6^ cfu of ATCC 6303. The blot was probed with mouse anti-SP0845^23–350^ sera and developed as described for panel A. The molecular mass marker (in kDa) is shown.

To test for the expression of SP0845 protein, lysates from 6 pneumococcal strains (D39, ATCC 6314, A66, ATCC 6301, ATCC 6303 and TIGR4) were resolved by SDS-PAGE and transferred to nitrocellulose membrane. The blot was probed with mouse anti-SP0845^23–350^ polyclonal sera (Fig. 4A). The immunoblot showed a prominent band of the expected size (~40 kDa) and comparable intensity in the 6 pneumococcal strains tested suggesting that SP0845 was expressed under *in vitro* culture conditions. Preimmune sera was non-reactive (data not shown).

In order to check whether SP0845 protein is expressed in *S*. *pneumoniae* during infection, BALB/c mice were intraperitoneally infected with 10^6^ cfu of ATCC 6303, 18 h later, pneumococci were isolated from the blood and spleen. Lysates were prepared from the isolated pneumococci and immunoblotted with polyclonal sera raised against SP0845^23–350^. The antisera identified a band of the expected size (~40 kDa; Fig. 4B). This data indicates that SP0845 is expressed by pneumococci during mice infection. Interestingly, unlike in the case of recombinant SP0845^23–350^ (Fig. 2), dimerization was not apparent in pneumococcal lysates. We do not understand the basis of this observation. It may be a result of the conditions used for purifying and/ or resolving SP0845^23–350^.

### SP0845 is surface exposed and accessible to antibodies

**Fig 5:**
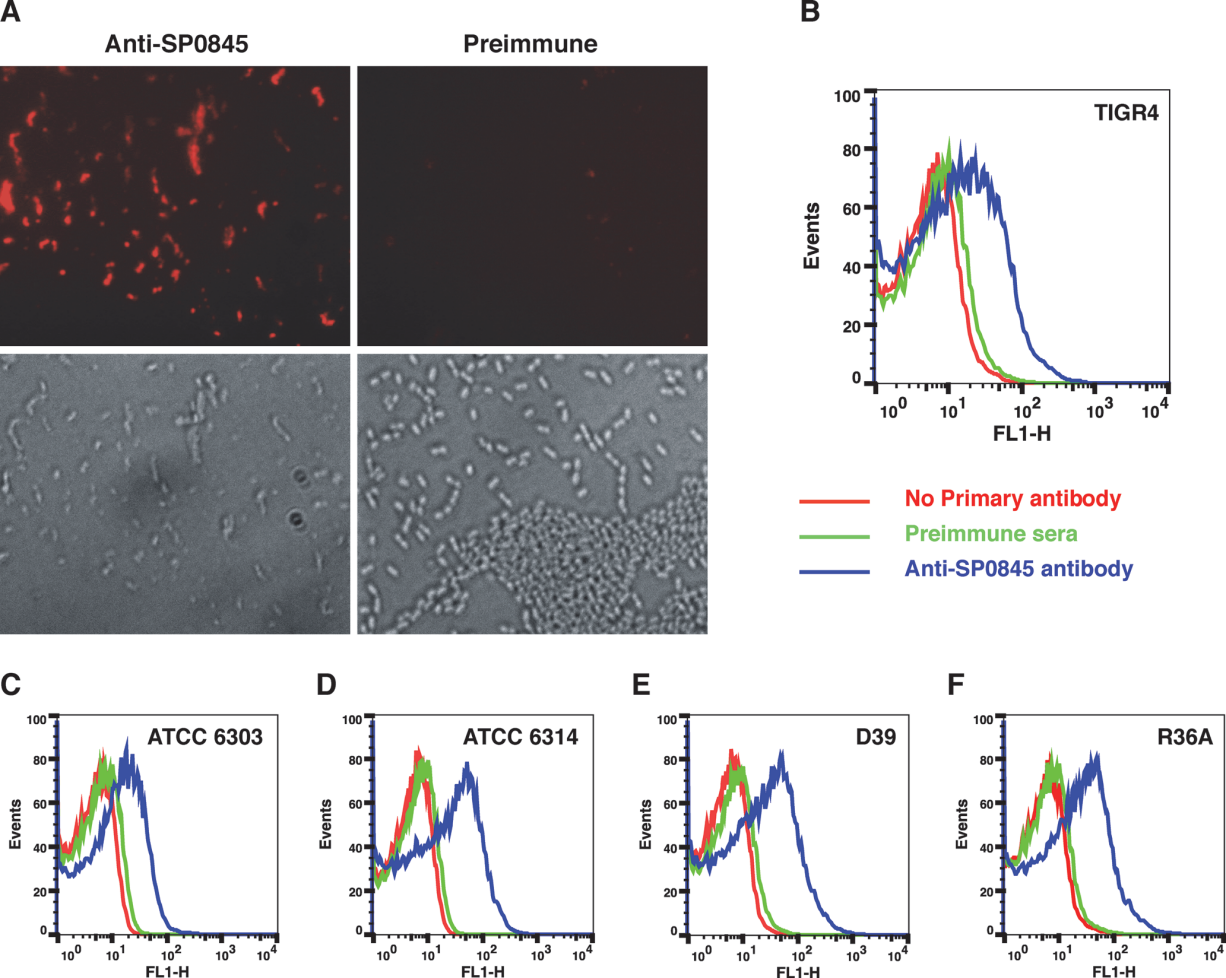
SP0845 is accessible to antibodies. (A) Mid-logarithmic phase TIGR4 cells were incubated with mouse anti-SP0845^23–350^ or mouse preimmune sera (negative control) followed by incubation with F(ab')_2_ fragment phycoerythrin-conjugated goat anti-mouse IgG + IgM (H + L) antibody. The processed samples were observed under a fluorescence microscope. The fluorescent images obtained with anti-SP0845^23–350^ and preimmune sera are in the upper left and right panels, respectively. The lower panels show the corresponding phase contrast images (magnification = 1000X). Surface expression of SP0845 was studied for pneumococcal strains TIGR4 (B), ATCC 6303 (C), ATCC 6314 (D), D39 (E) and R36A (F). Pneumococcal cells were incubated with either preimmune or anti-SP0845^23–350^ sera for 1 hr followed by a FITC-conjugated F(ab')_2_ fragment goat anti-mouse IgG + IgM (H + L) antibody as secondary antibody. The surface staining was detected by flow cytometry.

In order to confirm whether SP0845 is surface accessible, immunofluorescence microscopy was performed using mid-logarithmic phase TIGR4 and polyclonal sera raised against SP0845^23–350^. The data indicated that anti-SP0845^23–350^ polyclonal sera stained the pneumococcal cell surface whereas the preimmune serum was non-reactive (Fig. 5A).

Further, the surface expression of SP0845 (or its homologue) was assessed for 4 encapsulated strains TIGR4, ATCC 6303, ATCC 6314 and D39 by flow cytometry (Fig. 5B-E). The data demonstrated that SP0845 (or its homologue) was accessible to antibodies in the case of the 4 strains tested. Remarkably, the surface expression of SPD_0739 on D39 and its isogenic unencapsulated derivative R36A were comparable suggesting that the capsule did not physically hinder the binding of anti-SP0845 antibody to the surface of pneumococci (Fig. 5E and F).

### SP0845 induces high titer functional antibodies

SP0845^23–350^ formulated in alum was used to immunize 6–8 wk old female BALB/c mice. The end point titer of SP0845^23–350^ specific total IgG, IgG1, IgG2a, IgG2b and IgG3 in sera pooled from 12 immunized mice is shown in Table 2. The antigen-specific antibody endpoint titer data suggests that SP0845 is highly immunogenic and induces antibodies of various IgG subtypes.

**Table 2:** SP0845-specific serum antibody endpoint titers.

Ig class/ subclass	Endpoint titer^*a*^
Total IgG	6.0 × 10^5^
IgG1	6.9 × 10^4^
IgG2a	6.6 × 10^4^
IgG2b	1.0 × 10^5^
IgG3	1.0 × 10^4^

^*a*^ The SP0845-specific antibody endpoint titer was determined by ELISA after pooling serum from 12 mice immunized subcutaneously with recombinant SP0845. The endpoint titer was determined using two times the optical density obtained for the preimmune sera (diluted 1 in 100) as the cut off value.

**Fig 6:**
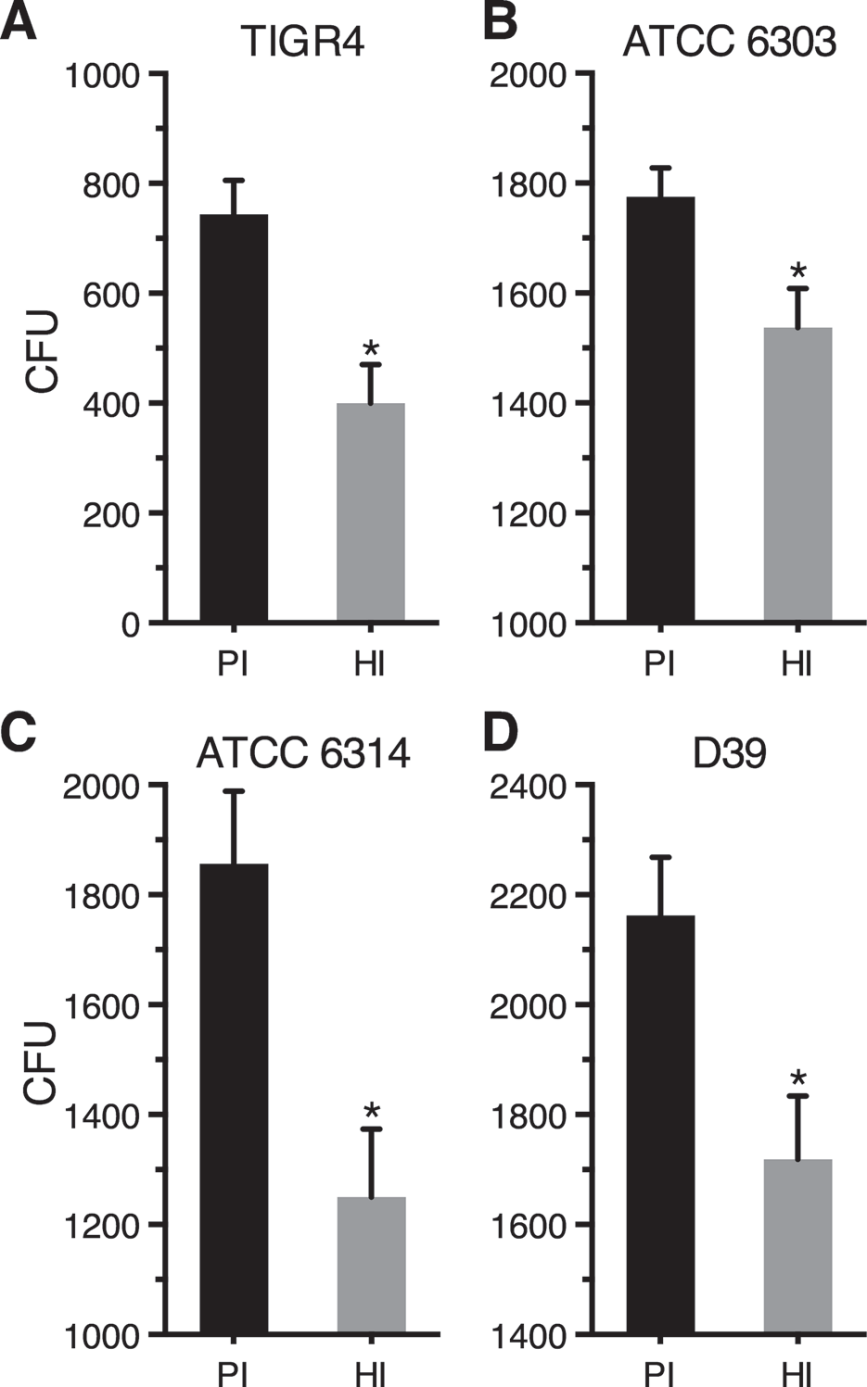
Anti-SP0845^23–350^ antibodies are functional as assessed by blood bactericidal assay. Pneumococcal cells (100–150 cfu) from strains TIGR4 (A), ATCC 6303 (B), ATCC 6314 (C) and D39 (D) were pretreated with either preimmune (PI, dark bar) or mouse anti-SP0845^23–350^ hyperimmune (HI, grey bar) sera and incubated with peripheral blood from CBA/N mice at 37°C for 3 h with rotation. The surviving bacteria were enumerated by plating serial dilutions on TSA plates in duplicate. The data represents the mean ± SD values. The data was analyzed using Student's unpaired t test.

To assess the functionality of antibodies directed against SP0845, a blood bactericidal assay was performed using peripheral blood from CBA/N mice. The ability of the hyperimmune sera raised against recombinant SP0845 to promote killing by mouse peripheral blood was determined for strains TIGR4, ATCC 6303, ATCC 6314 and D39 (Fig. 6). There was a 46% reduction in mean CFU values (hyperimmune compared to preimmune sera) for TIGR4 strain (Fig. 6A). The corresponding values for ATCC 6303, ATCC 6314 and D39 were 13, 32 and 20%, respectively (Fig. 6B-D). Based on Students' t test the *p* values obtained for strains TIGR4, ATCC 6303, ATCC 6314 and D39 are 0.0177, 0.0314, 0.0210 and 0.0284, respectively. There was a modest but statistically significant decrease in the number of surviving bacteria in the case of SP0845 hyperimmune sera treated pneumococci as compared to the sample treated with the preimmune sera in the 4 encapsulated strains analyzed clearly demonstrating that anti-SP0845 hyperimmune sera promotes killing of pneumococci by mouse blood *in vitro*.

### Immunization with recombinant SP0845 protects mice against intraperitoneal challenge with heterologous pneumococcal serotypes

**Fig 7:**
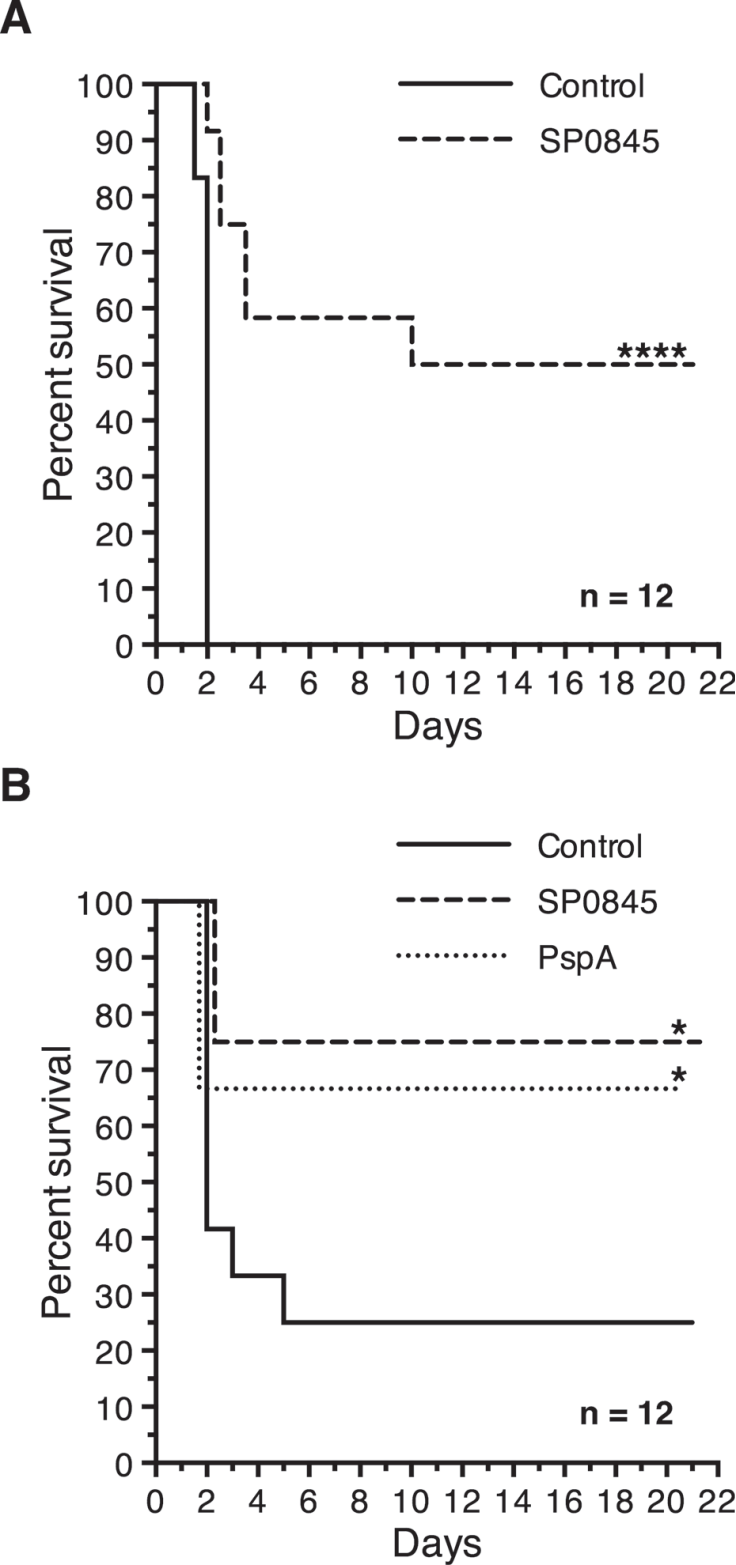
Immunization with recombinant SP0845 confers protection to mice against intraperitoneal challenge with heterologous pneumococcal strains. Groups of 12 BALB/c mice were immunized subcutaneously with recombinant SP0845^23–350^ (A) or SP0845^1–350^ or PspA^3–286^ from R36A in alum (B) or PBS formulated in alum (as control) followed by two booster doses on day 14 and 28. Mice were challenged intraperitoneally on day 35 with 10^5^ cfu of ATCC 6303 (A) or 5 x 10^6^ cfu of ATCC 6314 (B) and mice survival was monitored every 12 h for 21 days. Results presented here represent data from one experiment with 12 mice/group. Kaplan-Meier survival analysis (log rank test) was carried out to assess the statistical significance of the data.

To evaluate the ability of recombinant SP0845 to induce protective immunity, 12 BALB/c mice were immunized subcutaneously with SP0845^23–350^, SP0845^1–350^ or PspA^3–286^ in alum or PBS formulated in alum and challenged intraperitoneally with either ATCC 6303 (10^5^ cfu, Fig. 7A) or ATCC 6314 (5 x 10^6^ cfu, Fig. 7B). Among the animal set challenged with ATCC 6303, 50% (6 out of 12) of the mice in the SP0845^23–350^ immunized group survived for > 21 days whereas all the mice in the control who received saline with adjuvant died within 2 days (Fig. 7A). The median survival time of the SP0845^23–350^ immunized and control groups were 15.5 and 1.5 days, respectively (*p* < 0.001, log rank test). Similarly, in case of the set challenged with ATCC 6314, 75% (9 out of 12) of the SP0845^1–350^ immunized mice survived for > 21 days whereas only 25% (3 out of 12) of the mice in the control group were alive at day 21 (Fig. 7B). This was comparable to the data obtained with PspA^3–286^ (a well accepted protein vaccine candidate from *S*. *pneumoniae*) where 8 out of 12 (66.7%) mice survived > 21 days. Seven of the 12 (58.3%) mice in the control group succumbed to the infection within 48 h. The median survival time of the SP0845^1–350^ immunized and control groups in the set challenged with ATCC 6314 were 21 and 2 days, respectively (*p* < 0.05, log rank test; Fig. 7B). The active mouse protection experiments suggest that SP0845 is capable of inducing protective immunity in mice against intraperitoneal challenge with heterologous pneumococcal serotypes. Additionally, the protection conferred was comparable to that observed with PspA in the intraperitoneally challenge model used in our study.

## Discussion

Recent research on alternatives to the existing pneumococcal capsular polysaccharide-based vaccines has identified several protein vaccine candidates, such as Pneumolysin, PsaA, PspC, PcsK, StkP, SP0148 and PspA [5,15,18,27,28]. The objective of this study was to identify a novel highly conserved protein that could be a potential vaccine candidate. The immunoreactive proteins identified in this study included a choline binding protein, glycolytic enzymes, chaperone / proteins involved in post-translational modification, components of transporters and proteins involved in protein synthesis. The detection of several typically cytoplasmic metabolic enzymes e. g. glyceraldehyde-3-phosphate dehydrogenase and phosphoglycerate kinase on the cell surface of pneumococci is intriguing because none of those proteins contain apparent cell surface localization signal such as signal peptides, choline binding repeats or LPXTG motif. However, anchorless adhesins and invasins such as PavA and α enolase have been shown to be present on the surface of *S*. *pneumoniae* [60]. In addition, glycolytic enzymes glyceraldehyde-3-phosphate dehydrogenase and phosphoglycerate kinase were identified as cell wall associated proteins in an immunoproteomic screen performed using sera from children [26].

An ideal vaccine candidate should show minimal antigenic diversity across strains. Immune selection pressure not only results in epitope diversity but can also lead to the complete loss of the gene encoding the antigen. Our sequence analysis indicated that *sp0845* gene encodes a protein that is highly conserved (>98%) in the 36 pneumococcal strains (belonging to various serotypes) analyzed. For a vaccine antigen to be clinically useful, it must be ideally expressed by all the epidemiologically relevant strains. Immunoblot analysis revealed that SP0845 protein (or its homologue) is expressed in the 6 *in vitro* grown pneumococcal strains analyzed and also during infection in mice (Fig. 4). The observation that the antisera raised against SP0845 from TIGR4 cross-reacts with the 6 strains tested suggests conservation of SP0845 protein. None of the 36 pneumococcal strains analyzed in this study was found be deficient in SP0845 (or its homologue). It is noteworthy that DELTA-BLAST analysis of the non-redundant *H*. *sapiens* database did not reveal any human protein with significant amino acid identity (<11%) with SP0845. SP0845 cofractionated with PspA [61], phosphoglycerate kinase and glyceraldehyde-3-phosphate dehydrogenase [26], proteins that have been shown by other investigators to be present on the pneumococcal cell surface.

Bacterial ABC transporters are responsible for the uptake of a wide range of substrates including essential nutrients like sugars and metal ions. The availability of nutrients and/ or metal ions may have an impact on the fitness and/ or virulence of the bacterial pathogen. Our bioinformatic and functional analyses suggests that SPD_0739 is the substrate binding protein of an ABC transporter that is involved in the import of nucleosides with cytidine, uridine, guanosine and inosine as the preferred substrates. The deletion of *spd_0739* severely compromised pneumococcal virulence. Similar observations have been made for the substrate-binding protein of other pneumococcal ABC transporters. The manganese transporter component PsaA has been shown to be essential for virulence in different animal models of infection [62,63]. The branched-chain amino acid ABC transporter LivJ is necessary for disease pathogenesis [55].

Immunofluorescence microscopy showed that SP0845 is accessible to antibodies on pneumococci (Fig. 5A). Flow cytometry data further confirmed the surface accessibility of SP0845 (or its homologue) in the 4 encapsulated strains tested (Fig. 5B-E). This accessibility to antibodies was independent of the presence or absence of the capsule (Fig. 5E and F), an observation that needs to be confirmed using other strains. The accessibility of SP0845 (or its homologue) on the surface of different encapsulated strains is noteworthy as antibody-mediated opsonophagocytosis is the major mechanism by which pneumococci are cleared by the host [5,15,27,64]. The hyperimmune sera from mice immunized with recombinant SP0845 augmented killing of pneumococci in mouse blood (Fig. 6).

Immunization with recombinant SP0845 protected mice against intraperitoneal challenge with two heterologous virulent strains belonging to serotype 3 and 14 indicating that the conservation of the molecule across serotypes is biologically relevant. The finding that a functional antibody response was generated in the immunized mice suggested that the protection was, at least in part, mediated by antibodies. The degree of protection conferred by SP0845 and PspA was found to be comparable in our experimental setup.

In conclusion, our study demonstrates that SP0845 is a novel, highly conserved surface-accessible protein antigen and is expressed during infection. Protein database searches indicated that SP0845 does not share significant amino acid identity with any human protein. Our *in silico* and functional analyses suggests that SP0845 is the substrate-binding component of an ABC transporter involved in nucleoside transport. Deletion of the gene encoding the protein renders pneumococci avirulent. Immunization with recombinant SP0845 induced high titer antibodies that enhanced pneumococcal killing in a blood bactericidal assay. These data indicate that SP0845 is a potential vaccine candidate against *S*. *pneumoniae* infection.

## Supporting Information

S1 Fig The ligand-contacting residues of PnrA are conserved in SP0845. The alignment of the deduced protein sequence of full-length SP0845 with PnrA is shown. The numbers on the left and right of the sequence alignment refer to the amino acid position of the concern protein. The Cys residue responsible for coupling with the lipid moiety is highlighted in green. The ligand-contacting residues of PnrA when in complex with inosine (2FQW), guanosine (2FQX) and adenosine (2FQY) are in red font. The 12 ligand-contacting residues in PnrA are Asp 47, Ser 48, Phe 56, Asn 57, Asp 128, Met 178, Phe 206, Val 230, Gly 232, Val 257, Asp 258 and Lys 280. The ligand-contacting residues that are conserved and not conserved in SP0845 are indicated by an asterisk (*) and hash (#), respectively. Dash (-) represents missing amino acid residue.
(DOCX)

S2 Fig Expression of SPD_0739 (homologue of SP0845) in wildtype D39 and its derivatives. Lysates were prepared starting with equal number of wildtype (WT), *spd_0739* deficient (KO), *spd_0739* deficient strain genetically complemented with a plasmid expressing SPD_0739 (GC) and *spd_0739* deficient strain transformed with pDC123_DS (vector control; VC). The lysates were immunoblotted with either anti-SP0845 sera (A) or anti-PpmA sera (B) as the primary and horseradish peroxidase conjugated goat anti-mouse Ig antibody as the secondary antibody. Diaminobenzidine/ H_2_O_2_ was used as a substrate for the colour reaction. Molecular mass marker (in kDa) is shown to the left of each panel. (C) Surface expression of SPD_0739 in D39 and its derivative strains was analyzed by flow cytometry using anti-SP0845 sera. D39 treated with preimmune (PI) serum was used as the negative control. FITC conjugated F(ab')_2_ goat anti-mouse IgG + IgM (H + L) antibody was used as the secondary antibody. The data is presented as mean ± SD of geometric mean fluorescence intensity values (GMFI; n = 3). The data was analyzed using the one-way ANOVA with wildtype D39 treated with preimmune serum as the reference.
(DOCX)

S1 Table SP0845 alleles and allele frequency. (DOCX)
