# Supplementary material for: Conserved Surface Accessible Nucleoside ABC Transporter Component SP0845 Is Essential for Pneumococcal Virulence and Confers Protection In Vivo
Source: PLoS One. 2015 Feb 17;10(2):e0118154. doi: 10.1371/journal.pone.0118154 (PMC4331430; doi:10.1371/journal.pone.0118154)
Supplement: S1 Fig — The alignment of the deduced protein sequence of full-length SP0845 with PnrA is shown. The numbers on the left and right of the sequence alignment refer to the amino acid position of the concern protein. The Cys residue responsible for coupling with the lipid moiety is highlighted in green. The ligand-contacting residues of PnrA when in complex with inosine (2FQW), guanosine (2FQX) and adenosine (2FQY) are in red font. The 12 ligand-contacting residues in PnrA are Asp 47, Ser 48, Phe 56, Asn 57, Asp 128, Met 178, Phe 206, Val 230, Gly 232, Val 257, Asp 258 and Lys 280. The ligand-contacting residues that are conserved and not conserved in SP0845 are indicated by an asterisk (*) and hash (#), respectively. Dash (-) represents missing amino acid residue. (DOCX) [file pone.0118154.s001.docx]

*# **

SP0845 1 MNKKQWLGLGLVAVAAVGLAACGN-RSSR-NAASSSDVKTKAAIVTDTGGVDDKSFNQSA 58

2FQW 1 MREKWVRAFAGVFCAMLLIGCSKSDRPQMGNAGGAEGGDFVVGMVTDSGDIDDKSFNQQV 60

2FQX 1 MREKWVRAFAGVFCAMLLIGCSKSDRPQMGNAGGAEGGDFVVGMVTDSGDIDDKSFNQQV 60

2FQY 1 MREKWVRAFAGVFCAMLLIGCSKSDRPQMGNAGGAEGGDFVVGMVTDSGDIDDKSFNQQV 60

SP0845 59 WEGLQAWGKEHNLSKDNGFTYFQSTSEADYANNLQQAAGS-YNLIFGVGFALNNAVKDAA 117

2FQW 61 WEGISRFAQENNAK----CKYVTASTDAEYVPSLSAFADENMGLVVACGSFLVEAVIETS 116

2FQX 61 WEGISRFAQENNAK----CKYVTASTDAEYVPSLSAFADENMGLVVACGSFLVEAVIETS 116

2FQY 61 WEGISRFAQENNAK----CKYVTASTDAEYVPSLSAFADENMGLVVACGSFLVEAVIETS 116

*

SP0845 118 KEHTDLNYVLIDDVIKDQKNVASVTFADNESGYLAGVAAAKTTKTK---QVGFVGGIESE 174

2FQW 117 ARFPKQKFLVIDAVVQDRDNVVSAVFGQNEGSFLVGVAAALKAKEAGKSAVGFIVGMELG 176

2FQX 117 ARFPKQKFLVIDAVVQDRDNVVSAVFGQNEGSFLVGVAAALKAKEAGKSAVGFIVGMELG 176

2FQY 117 ARFPKQKFLVIDAVVQDRDNVVSAVFGQNEGSFLVGVAAALKAKEAGKSAVGFIVGMELG 176

# * * *

SP0845 175 VISRFEAGFKAGVASVDPSIKVQVDYAGSFGDAAKGKTIAAAQYAAGADIVYQVAGGTGA 234

2FQW 177 MMPLFEAGFEAGVKAVDPDIQVVVEVANTFSDPQKGQALAAKLYDSGVNVIFQVAGGTGN 236

2FQX 177 MMPLFEAGFEAGVKAVDPDIQVVVEVANTFSDPQKGQALAAKLYDSGVNVIFQVAGGTGN 236

2FQY 177 MMPLFEAGFEAGVKAVDPDIQVVVEVANTFSDPQKGQALAAKLYDSGVNVIFQVAGGTGN 236

** *

SP0845 235 GVFAEAKSLNESRPENEKVWVIGVDRDQEAEGKYTSKDGKESNFVLVSTLKQVGTTVKDI 294

2FQW 237 GVIKEAR---DRRLNGQDVWVIGVDRDQYMDGVY---DGSKS-VVLTSMVKRADVAAERI 289

2FQX 237 GVIKEAR---DRRLNGQDVWVIGVDRDQYMDGVY---DGSKS-VVLTSMVKRADVAAERI 289

2FQY 237 GVIKEAR---DRRLNGQDVWVIGVDRDQYMDGVY---DGSKS-VVLTSMVKRADVAAERI 289

SP0845 295 SNKAERGEFPGGQVIVYSLKDKGVDLAVTN--LSEEGKKAVEDAKAKILDGSVKVPEK-- 350

2FQW 290 SKMAYDGSFPGGQSIMFGLEDKAVGIPEENPNLSSAVMEKIRSFEEKIVSKEIVVPVRSA 349

2FQX 290 SKMAYDGSFPGGQSIMFGLEDKAVGIPEENPNLSSAVMEKIRSFEEKIVSKEIVVPVRSA 349

2FQY 290 SKMAYDGSFPGGQSIMFGLEDKAVGIPEENPNLSSAVMEKIRSFEEKIVSKEIVVPVRSA 349

SP0845 351 ---- 350

2FQW 350 RMMN 353

2FQX 350 RMMN 353

2FQY 350 RMMN 353

**Figure S1. The ligand-contacting residues of PnrA are conserved in SP0845.** The alignment of the deduced protein sequence of full-length SP0845 with PnrA is shown. The numbers on the left and right of the sequence alignment refer to the amino acid position of the concern protein. The Cys residue responsible for coupling with the lipid moiety is highlighted in green. The ligand-contacting residues of PnrA when in complex with inosine (2FQW), guanosine (2FQX) and adenosine (2FQY) are in red font. The 12 ligand-contacting residues in PnrA are Asp 47, Ser 48, Phe 56, Asn 57, Asp 128, Met 178, Phe 206, Val 230, Gly 232, Val 257, Asp 258 and Lys 280. The ligand-contacting residues that are conserved and not conserved in SP0845 are indicated by an asterisk (*) and hash (#), respectively. Dash (-) represents missing amino acid residue.
