# Supplementary material for: Conserved Surface Accessible Nucleoside ABC Transporter Component SP0845 Is Essential for Pneumococcal Virulence and Confers Protection In Vivo
Source: PLoS One. 2015 Feb 17;10(2):e0118154. doi: 10.1371/journal.pone.0118154 (PMC4331430; doi:10.1371/journal.pone.0118154)
Supplement: S2 Fig — Lysates were prepared starting with equal number of wildtype (WT), spd_0739 deficient (KO), spd_0739 deficient strain genetically complemented with a plasmid expressing SPD_0739 (GC) and spd_0739 deficient strain transformed with pDC123_DS (vector control; VC). The lysates were immunoblotted with either anti-SP0845 sera (A) or anti-PpmA sera (B) as the primary and horseradish peroxidase conjugated goat anti-mouse Ig antibody as the secondary antibody. Diaminobenzidine/ H2O2 was used as a substrate for the colour reaction. Molecular mass marker (in kDa) is shown to the left of each panel. (C) Surface expression of SPD_0739 in D39 and its derivative strains was analyzed by flow cytometry using anti-SP0845 sera. D39 treated with preimmune (PI) serum was used as the negative control. FITC conjugated F(ab')2 goat anti-mouse IgG + IgM (H + L) antibody was used as the secondary antibody. The data is presented as mean ± SD of geometric mean fluorescence intensity values (GMFI; n = 3). The data was analyzed using the one-way ANOVA with wildtype D39 treated with preimmune serum as the reference. (DOCX) [file pone.0118154.s002.docx]

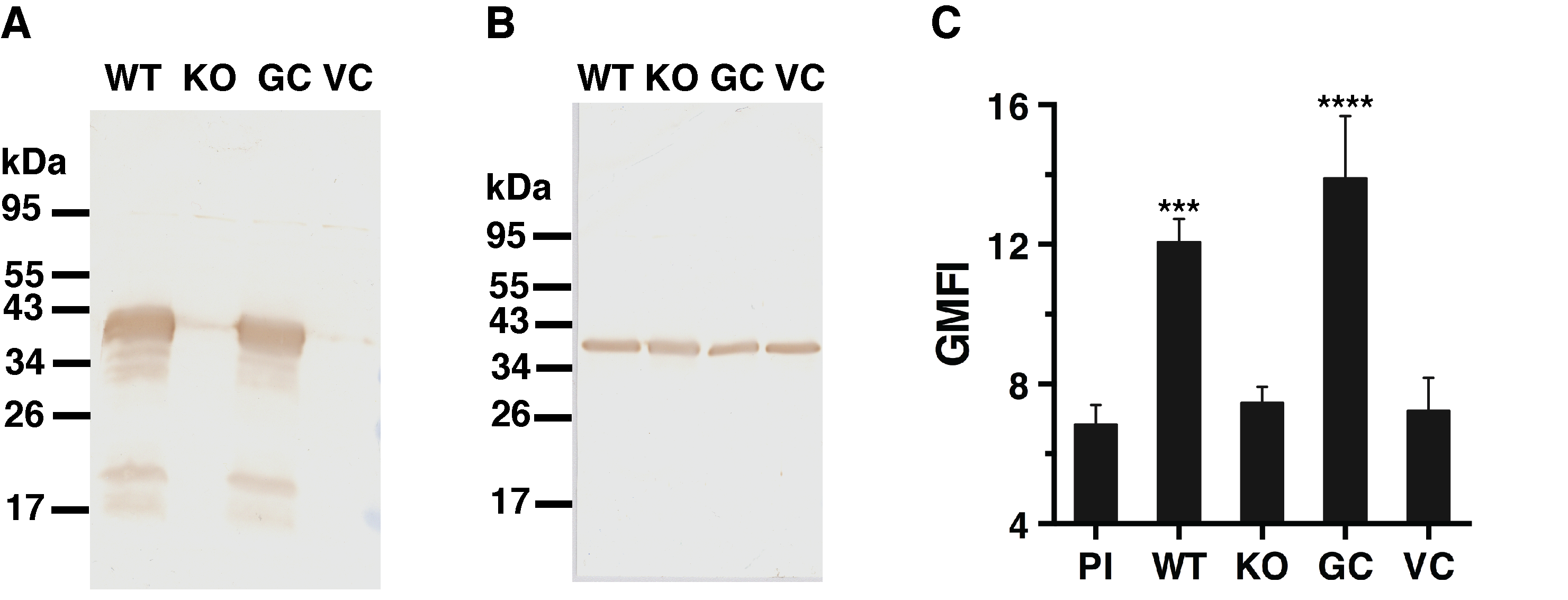


**Figure S2. Expression of SPD_0739 (homologue of SP0845) in wildtype D39 and its derivatives.** Lysates were prepared starting with equal number of wildtype (WT), *spd_0739* deficient (KO), *spd_0739* deficient strain genetically complemented with a plasmid expressing SPD_0739 (GC) and *spd_0739* deficient strain transformed with pDC123_DS (vector control; VC). The lysates were immunoblotted with either anti-SP0845 sera (A) or anti-PpmA sera (B) as the primary and horseradish peroxidase conjugated goat anti-mouse Ig antibody as the secondary antibody. Diaminobenzidine/ H_2_O_2_ was used as a substrate for the colour reaction. Molecular mass marker (in kDa) is shown to the left of each panel. (C) Surface expression of SPD_0739 in D39 and its derivative strains was analyzed by flow cytometry using anti-SP0845 sera. D39 treated with preimmune (PI) serum was used as the negative control. FITC conjugated F(ab')_2_ goat anti-mouse IgG + IgM (H + L) antibody was used as the secondary antibody. The data is presented as mean ± SD of geometric mean fluorescence intensity values (GMFI; n = 3). The data was analyzed using the one-way ANOVA with wildtype D39 treated with preimmune serum as the reference.
