# Supplementary material for: Conserved Surface Accessible Nucleoside ABC Transporter Component SP0845 Is Essential for Pneumococcal Virulence and Confers Protection In Vivo
Source: PLoS One. 2015 Feb 17;10(2):e0118154. doi: 10.1371/journal.pone.0118154 (PMC4331430; doi:10.1371/journal.pone.0118154)
Supplement: S1 Table — (DOCX) [file pone.0118154.s003.docx]

**Table S1**

SP0845 alleles and allele frequency.

| **Allele^a^** | **Accession Number/ Gene ID** | **Representative pneumococcal strain** | **Relative allele frequency (%)** |
| --- | --- | --- | --- |
| 1 | AAK74976 | TIGR4 | 1 (2.78) |
| 2 | EDK71937 | SP19-BS75 | 1 (2.78) |
| 3 | EDT91557 | CDC1087-00 | 1 (2.78) |
| 4 | HM775954, 169833858, 237650835, 237821560, ACA36972, ACB90039, ACO17165, ACO20763, EDK64959, EDK68220, EDK73033, EDK75474, EDK79841, EDK81156, EDT92304, EDT95233, EDT97431, HM775956, HM775957, HM775958 | ATCC 6301 | 20 (55.55) |
| 5 | HM775959, EDT50146 | ATCC 6323 | 2 (5.55) |
| 6 | ABJ55247, AAK99551 | D39 | 2 (5.55) |
| 7 | HM775960, ACF54929, ACO19030, ACO23042, CAR68606, EDK63820, EDT99089, EES38713 | ATCC 6326 | 8 (22.22) |
| 8 | HM775955 | ATCC 6303 | 1 (2.78) |

^a^ The amino acid sequences of the mature SP0845 protein (residues 22-350) from the 36 pneumococcal strains were aligned using MacVector. The alleles were identified based on the amino acid sequence and assigned allele names (1 through 8) arbitrarily.
